# HIF–VEGF Pathways Are Critical for Chronic Otitis Media in *Junbo* and *Jeff* Mouse Mutants

**DOI:** 10.1371/journal.pgen.1002336

**Published:** 2011-10-20

**Authors:** Michael T. Cheeseman, Hayley E. Tyrer, Debbie Williams, Tertius A. Hough, Paras Pathak, Maria R. Romero, Helen Hilton, Sulzhan Bali, Andrew Parker, Lucie Vizor, Tom Purnell, Kate Vowell, Sara Wells, Mahmood F. Bhutta, Paul K. Potter, Steve D. M. Brown

**Affiliations:** 1 MRC Mammalian Genetics Unit, MRC Harwell, Harwell, United Kingdom; 2 Mary Lyon Centre, MRC Harwell, Harwell, United Kingdom; 3 Nuffield Department of Surgical Sciences, University of Oxford, Oxford, United Kingdom; Tel Aviv University, Israel

## Abstract

Otitis media with effusion (OME) is the commonest cause of hearing loss in children, yet the underlying genetic pathways and mechanisms involved are incompletely understood. Ventilation of the middle ear with tympanostomy tubes is the commonest surgical procedure in children and the best treatment for chronic OME, but the mechanism by which they work remains uncertain. As hypoxia is a common feature of inflamed microenvironments, moderation of hypoxia may be a significant contributory mechanism. We have investigated the occurrence of hypoxia and hypoxia-inducible factor (HIF) mediated responses in *Junbo* and *Jeff* mouse mutant models, which develop spontaneous chronic otitis media. We found that *Jeff* and *Junbo* mice labeled *in vivo* with pimonidazole showed cellular hypoxia in inflammatory cells in the bulla lumen, and in *Junbo* the middle ear mucosa was also hypoxic. The bulla fluid inflammatory cell numbers were greater and the upregulation of inflammatory gene networks were more pronounced in *Junbo* than *Jeff*. *Hif-1α* gene expression was elevated in bulla fluid inflammatory cells, and there was upregulation of its target genes including Vegfa in *Junbo* and *Jeff*. We therefore investigated the effects in *Junbo* of small-molecule inhibitors of VEGFR signaling (PTK787, SU-11248, and BAY 43-9006) and destabilizing HIF by inhibiting its chaperone HSP90 with 17-DMAG. We found that both classes of inhibitor significantly reduced hearing loss and the occurrence of bulla fluid and that VEGFR inhibitors moderated angiogenesis and lymphangiogenesis in the inflamed middle ear mucosa. The effectiveness of HSP90 and VEGFR signaling inhibitors in suppressing OM in the *Junbo* model implicates HIF–mediated VEGF as playing a pivotal role in OM pathogenesis. Our analysis of the *Junbo* and *Jeff* mutants highlights the role of hypoxia and HIF–mediated pathways, and we conclude that targeting molecules in HIF–VEGF signaling pathways has therapeutic potential in the treatment of chronic OM.

## Author Summary

Otitis media with effusion (OME) is the commonest cause of hearing loss in children, and treatment using grommets remains the commonest surgical procedure in children. Chronic forms of OM are known from human population studies to have a significant genetic component, but little is known of the underlying genes or pathways involved. We have analyzed two chronic OM mouse models, the *Junbo* and *Jeff* mutants, and have found that both demonstrate hypoxia and hypoxia-inducible factor (HIF) mediated responses. There is upregulation of inflammatory pathways in the mutant middle ears and in *Junbo* elevation of cytokines that modulate Hif-1α. Hif-1α levels are raised in the middle ear as well as downstream targets of HIF such as Vegfa. We explored the effects of small-molecule inhibitors of HSP90 and VEGF receptor signaling in the *Junbo* mutant and found significant reductions in hearing loss, the occurrence of bulla fluid, and moderation of vascular changes in the inflamed middle ear mucosa with the VEGF receptor inhibitors. The study of the *Junbo* and *Jeff* mutants demonstrates the role of hypoxia and HIF mediated pathways in OM pathogenesis, and it indicates that targeting the HIF–VEGF pathway may represent a novel approach to therapeutic intervention in chronic OM.

## Introduction

Chronic middle ear effusion without the symptoms of acute infection is termed otitis media (OM) with effusion and can be sequel to acute bacterial otitis media. Otitis media with effusion (OME) is the most common cause of hearing impairment in children potentially causing language delays, learning and behavioral problems [1], [2]. About 2.2 million episodes of OME occur annually in the US with an annual cost estimate of $4.0 billion [3].The prolonged ventilation of the middle ear with tympanostomy tubes, also known as grommets, remains the best treatment for OME [4]. Placement of tympanostomy tubes is the most common operation in the UK (30,000 procedures per annum) however the mechanism by which they work remains uncertain. As hypoxia is a common feature of inflamed microenvironments [5], [6] the therapeutic benefits of ventilating the middle ear may conceivably include the moderation of hypoxia as well as relieving negative pressure and fluid drainage.

Responses to hypoxia are mediated by Hypoxia Inducible Factor (HIF) protein a transcription factor that induces genes whose products restore blood supply, nutrients and energy production to maintain tissue homeostasis. Constitutively expressed HIF-1α is modified by prolyl hydroxylase domain (PHD) enzymes under normoxic conditions and targeted for proteasomal degradation. Under hypoxic conditions PHD activity is limited and HIF-1α is stabilized and forms a heterodimer with HIF-1β before translocation to the nucleus where it binds to hypoxic response elements [7]. HIF signaling is also regulated by inflammation at the transcriptional level via HIF-1α interactions with the master regulator of inflammation NF-κB [8]–[10] and at the translational level by cytokines such as IL-1β and TNF-α [5], [6]. HIF responses are adaptive and help overcome localized ischemia as well as regulating innate immune responses to microbial infections [11] but chronic hypoxic inflammation may result in dysregulated HIF signaling and lead to pathological outcomes. Examples include fibrosis via immune cell activation [6] and the progression of rheumatoid arthritis [12] via angiogenesis caused by HIF-induced vascular endothelial growth factor (VEGF). Indeed, treatment using VEGF receptor (VEGFR) signaling inhibitors moderates experimentally-induced arthritis [13].

Although hypoxia might be expected in the inflammatory conditions of chronic OM the evidence is inconsistent. Some studies have found that OME fluids in the middle ear cavity (bulla) have oxygen tensions similar to venous blood, of ∼40 Torr [14], [15]. Another study reported pO_2_ in mucoid and serous OME fluids were lower ∼29–32 Torr. However, these values were not significantly different than pO_2_ values in barotrauma bulla fluids [16]. Nevertheless, there are a few studies to suggest that the downstream HIF signaling protein VEGF plays a role in experimental and clinical OME. Injection of recombinant VEGF into the rat bulla causes fluid effusion, mucosal inflammation and an increase in vascular permeability [17]. Vegf, Vegfr1 (also known as Flt1) and Vegfr2 (also known as Kdr) gene and protein expression are upregulated in the endotoxin-induced rat model of OME [18], [19] and Vegf protein is elevated in mouse middle and inner ear tissue after challenge with *Haemophilus influenzae* [20]. Moreover VEGF mRNA and protein are detectable in bulla fluids of patients with OME [18], [21]. However, these studies have not investigated the role of hypoxia and HIF signaling in the inflamed middle ear.

There is a significant genetic component predisposing to recurrent or chronic OM in human populations [22]–[26]. However, while a number of association studies have been carried out, sample sizes are relatively small and confirmation will be required through larger scale analyses and replication. A number of underlying OM susceptibility genes have been discovered in the mouse which represents a powerful model for dissecting the underlying pathways. These genes apppear to fall into three categories; those which are involved in craniofacial development and thereby Eustachian tube morphology and function, TLR4/MyD88 pathway genes that regulate innate immune function, and TGF-β pathway genes that modulate pro-inflammatory responses [27]. The two OM mouse mutants *Junbo* and *Jeff*, generated by *N′*-ethyl-*N′*-nitrosourea mutagenesis, represent powerful models for human OM as unlike many other mouse mutants they are non-syndromic and do not show the wide-ranging pleiotropic effects often associated with middle ear inflammatory disease in other models [27]. *Jeff* encodes a mutation in the Fbxo11 protein [28] and *Junbo* encodes a mutation in the transcription factor Evi1 [29]. Heterozygote *Junbo* (*Jbo/+*) and *Jeff* (*Jf/+*) mice develop OM spontaneously in the absence of other organ pathology or overt immune deficiency [28], [29]. There is an association between polymorphisms in *FBXO11*, the human homologue of the *Jeff* mutant protein, in OME and recurrent OM [24] and severe OM [25], but there is no such association with *EVI1* polymorphisms.

In this work we have analyzed the two OM mouse models *Junbo* and *Jeff* by hypoxia labeling, transcriptional profiling and, in *Junbo*, using small-molecule inhibitors. We have discovered that the response to chronic inflammatory hypoxia via Hif-1α signaling and VEGF pathways is critical for chronic OM. Our analysis of the two mutants provides insight into the molecular and genetic mechanisms of OM and identifies potential new therapeutic targets for OM.

## Results

### Hypoxia in the middle ear

**Figure 1:**
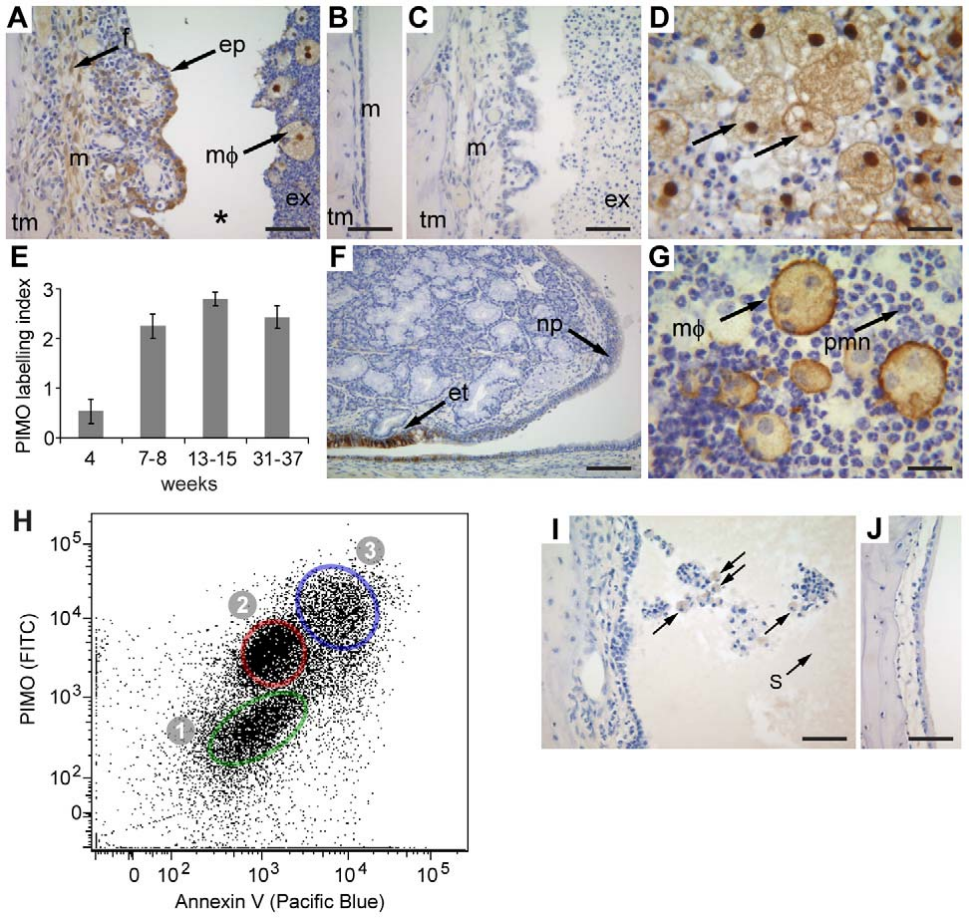
The inflamed middle ear of the *Jbo/+* and *Jf/+* mice is hypoxic. (A) *Jbo/+* mouse labeled with pimonidazole (PIMO), arrows indicate hypoxia in [ep] epithelium, [f] connective tissue fibrocyte, [mΦ] foamy macrophage; [tm] temporomandibular bone, [m] thickened inflamed mucosa, [ex] exudate. Note * the cleft is an artifact produced by tissue processing. (B) The normal thin mucosa [m] is not labeled in *Junbo* wild type (+/+) mice. (C) An unlabeled *Jbo/+* mouse is a negative control for anti-PIMO antibody. (D) Hypoxia in foamy mΦ. (E) The middle ear in *Jbo/+* mice is chronically hypoxic. The labeling index scores one point each for PIMO-positive staining in inflammatory cells in the bulla; mucosal epithelium; and mucosal connective tissues. Histogram bars are mean ± SEM. 4 wk group size *n* = 8, 7–8 wk *n* = 10, 13–15 wk *n* = 5, 31–37 wk *n* = 7. (F) The Eustachian tube epithelium [et] is hypoxic in a +/+ mouse but the adjacent nasopharynx epithelium [np] is normoxic. (G) Bulla fluid cytology from a *Jbo/+* mouse shows F4/80 foamy mΦ and polymorphonuclear cells (PMN). (H) FACS analysis of PIMO-labeled *Jbo/+* bulla fluids stained with Ly6G and Ly6C (PMN marker), for PIMO (hypoxia), and Annexin V (apoptosis marker). The PMN population was gated on the Ly6G and Ly6C signal. Population (1) normoxic viable PMN, (2) hypoxic viable PMN, (3) hypoxic apoptotic PMN. (I) *Jf/+* mouse PIMO labeling was restricted to inflammatory cells in the bulla fluids and there was no detectable mucosal labeling, (J) *Jeff* +/+ mouse does not show PIMO labeling. Scale bars: A,B,C,I,J = 50 µm; D,G =  20 µm; F = 100 µm.

We surmised that the inflamed microenvironment in chronic OM is hypoxic and proceeded to test this hypothesis by the analysis of the *Junbo* and *Jeff* mutants. To test whether the inflammatory cells that accumulate within the middle ear were hypoxic we injected mice *in vivo* with pimonidazole (PIMO), a marker that labels cells and tissues with a pO_2_<10 Torr (∼1.5% O_2_). FACS analysis revealed hypoxia in viable and apoptotic polymorphonuclear cell (PMN) populations in the purulent bulla fluids of *Jbo/+* (7.1±1.7×10^6^ cells per µl, *n* = 10) and serous effusions of *Jf/+* mice (55±25×10^3^ cells per µl, *n* = 5) (Figure 1H and Table 1). In addition, immunohistochemistry showed hypoxia in F4/80-positive foamy macrophages (mΦ) within the bulla, the epithelium and in the connective tissues of the thickened, inflamed middle ear mucosa of *Jbo/+* mice (Figure 1A, 1C, 1D, 1G) but not in the normal thin mucosa of wild type (+/+) mice (Figure 1B). Hypoxia was evident at 4 wk, increased at 7–8 wk and remained chronically elevated for >30 wk (Figure 1E). The only part of the tubotympanum that appeared hypoxic under normal physiological conditions was the Eustachian tube (Figure 1F). In *Jf/+* mice PIMO labeling was restricted to inflammatory cells in the bulla fluids and there was no detectable mucosal labeling (Figure 1I, 1J).

**Table 1:** Viable and apoptotic PMN populations in bulla fluids of *Jbo/+* and *Jf/+* mice are hypoxic.

			PMN		hypoxic PMN		normoxic PMN	
	age	*n*	viable	apoptotic	viable	apoptotic	viable	apoptotic
*Jbo/+*	5–8 wk	7	61±4	21±2	12±4	49±8	79±5	45±5^b^
	12–17 wk	13	67±5	15±2	22±6	85±3^a^	63±6	11±2
*Jf/+*	7–11 wk	12	52±7	40±8	45±12	16±2	47±12	83±2

Populations are expressed as percentages of their parent viable or apoptotic populations. The population of hypoxic apoptotic PMN was larger (^a^ *P* = 0.0024) in 12–17 wk *Jbo/+* than in 5–8 wk *Jbo/+* mice and conversely, the normoxic apoptotic PMN population was larger (^b^ *P* = 0.00027) in 5–8 wk *Jbo/+* than 12–17 wk *Jbo/+* mice. Propidium iodide staining showed that 7±2% of the *Jbo/+* and 8±2% of the *Jf/+* PMN populations were necrotic. Mean polymorphonuclear cell (PMN) percentages ± SEM. Statistics were performed using 2-tailed Student t-tests.

### Chronic inflammatory hypoxia and upregulation of HIF and VEGF pathways

*Evi1* and *Fbxo11* were expressed in the inflammatory cells that accumulate within the bulla fluids of *Jbo/+* and *Jf/+* mice, but only *Evi1* (23–37 fold) was expressed at higher levels relative to a normoxic baseline control of *Jbo/+* or *Jf/+* venous blood white blood cells (WBC). The Evi1 target genes *Jun* (28–50 fold) and *Fos* (5–10 fold) were also elevated in *Jbo/+* and *Jf/+* bulla fluid inflammatory cells relative to blood WBC (Figure S1).

We found elevated expression of *Hif-1α* (6–12 fold) and HIF responsive genes *Vegf*a (41–122 fold) and Slc2a1 (also known as *Glut1*) (8 fold) in *Jbo/+* and *Jf/+* bulla fluid WBC relative to blood WBC (Figure S1) and Vegf signaling arrays showed elevated expression in a wide spectrum of Vegf pathway genes (Table S1). In *Jbo/+* and *Jf/+* mice we obtained data for 84 and 77 genes respectively and there was a strong similarity in pattern of upregulation of genes belonging to functional groups such as Vegf/growth factors and their receptors, Akt and Pi-3-Kinases, phospholipases A2, heat shock proteins, *Hif-1α* and *Arnt* (*Hif-1β*). 44% of the genes were significantly elevated (>2-fold, *P*<0.05) in both mutants; 18% genes were elevated in both mutants with levels in either *Jf/+* or *Jbo/+* achieving statistical significance; 4% of genes were elevated in both mutants but *P*>0.05, and 8% of genes were up-regulated in *Jbo/+* mice but beneath detection limits for *Jf/+*. 11% of genes were unaltered in one or other mutant; 14% unaltered in both mutants and only 1 gene was significantly lower in both mutants (Table S1, Figure S2).

**Figure 2:**
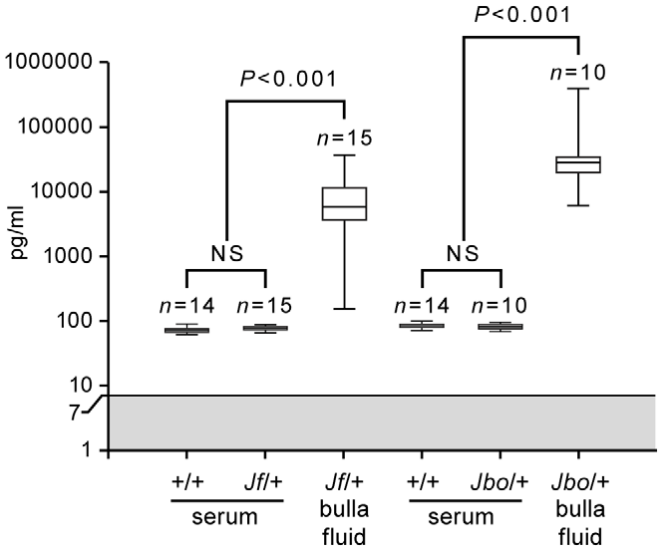
Vegfa titers are elevated in bulla fluids of 8-week-old *Jbo/+* and *Jf/+* mice compared to serum. Protein titers of Vegfa in serum and bulla fluids of 8 wk *Junbo* (+/+ and *Jbo/+*) and *Jeff* (+/+ and *Jf/+*) mice. The gray zone represents the lowest assay standard (7 pg/ml). Each box represents the median with 25 and 75% inter-quartile ranges, with whiskers representing the data range (minimum and maximum). A Kruskall-Wallis test was performed followed by Dunn's multiple comparison tests for post hoc testing.

In 8 wk old mice, Vegfa protein was elevated ∼74-fold in *Jf/+* bulla fluids compared with *Jf/+* sera (median values of 5,793 pg/ml versus 78 pg/ml; *P*<0.001) and ∼335-fold in *Jbo/+* bulla fluids compared with *Jbo/+* sera (median values of 28,123 pg/ml versus 84 pg/ml; *P*<0.001) (Figure 2). The difference between Vegfa titers in *Jf/+* and *Jbo/+* bulla fluids did not achieve statistical significance, nor did titer differences between *Jeff* and *Junbo* mutant and wild type (+/+) sera (Kruskall Wallis ANOVA and Dunn's multiple comparison post hoc tests).

Using inflammation arrays we obtained data for 84 genes in *Jbo/+* and 79 genes in *Jf/+* mice. Again there was a strong similarity in the pattern of upregulation of gene expression for chemokines, cytokines, their receptors and acute phase response mediators. 35% of genes were significantly elevated (>2-fold, *P*<0.05) in both mutants; 31% genes were elevated in both mutants with either *Jf/+* or *Jbo/+* achieving statistical significance; 11% of genes were elevated in both mutants but did not achieve statistical significance (*P*>0.05); 6% of genes were up-regulated in *Jbo/+* mice but beneath detection limits for *Jf/+*. 10% of genes were unaltered in one or other mutant, 5% unaltered in both and only 2 genes were significantly lower (>2-fold, *P*<0.05) in one or both mutants (Table S2, Figure S3).

**Figure 3:**
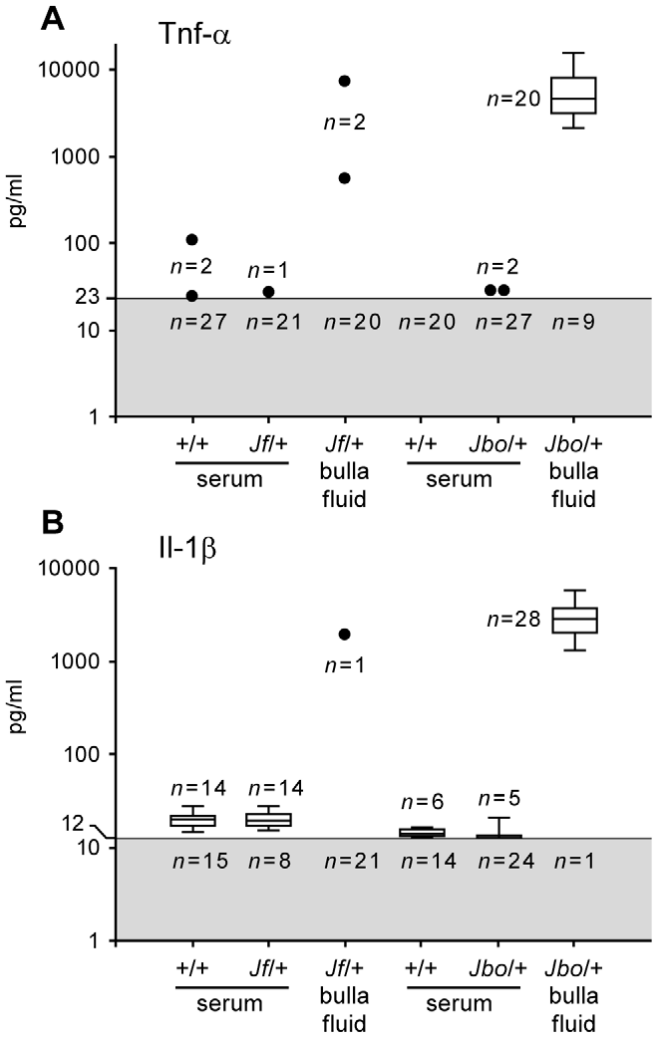
Il-1β and Tnf-α titers in bulla fluids are elevated in *Jbo/+* but not *Jf/+* mice. Protein titers of (A) Tnf-α and (B) Il-1β in serum and bulla fluids of 8 wk *Junbo* (+/+ and *Jbo/+*) and *Jeff* (+/+ and *Jf/+*) mice. Figures in the gray zone represent the numbers of samples with values beneath the lowest assay standard (23 pg/ml for Tnf-α and 12 pg/ml for Il-1β. Each box and whisker symbol represents the minimum, 25% quartile, median, 75% quartile and maximum for reportable measurements while single values are represented by dots. Since the level of Tnf-α and Il-1β in many *Jf/+* and *Jbo/+* sera and *Jf/+* bulla fluid samples was beneath detection limits, a statistical analysis was not performed.

Il-1β and Tnf-α are known modulators of Hif-1α translation and array data indicated that they were significantly elevated (*P*<0.05) in *Jbo/+* (Il-1β 26-fold; Tnf-α 78-fold) but elevations in *Jf/+* expression (Il-1β 3-fold; Tnf-α 50-fold) were not statistically significant (Table S2). We therefore went on to determine their protein titers. Il-1β and Tnf-α were elevated in *Jbo/+* bulla fluid but not consistently so in *Jf/+* mice (Figure 3). Two of 22 *Jf/+* mice had Tnf-α bulla fluid titers of 571 and 7,352 pg/ml respectively whereas 20/29 *Jbo/+* mice had a median bulla fluid titer of 4,598 pg/ml (range 2,156 to 15,293 pg/ml). The Tnf-α serum titers for mutant and +/+ mice were comparable and ranged from 24 to 107 pg/ml. One of 22 *Jf/+* mice had an Il-1β bulla fluid titer of 1920 pg/ml whereas 28/29 *Jbo/+* mice had a median bulla fluid titer of 2,862 pg/ml (range 1,319 to 5,819 pg/ml). The Il-1β serum titers for mutant and +/+ mice were comparable and ranged from 15 to 27 pg/ml Figure 3).

### Inhibitors of VEGFR signaling and HSP90 moderate hearing loss, angiogenesis, and lymphangiogenesis in *Junbo* mice

**Figure 4:**
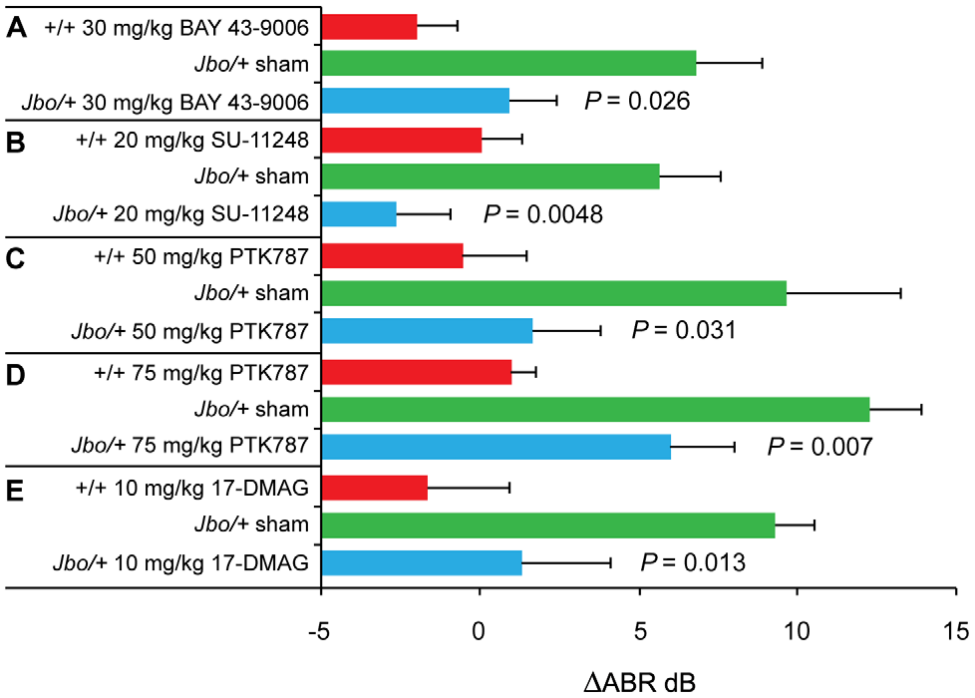
Treatment of *Jbo/+* mice with VEGF receptor inhibitors and the HSP90 inhibitor 17-DMAG moderates hearing loss. (A) Change in Auditory Brain Stem response (ΔABR) in decibels (dB) in 15 d treatment with BAY 43-9006 (+/+, sham *Jbo/+*, drug *Jbo/+ n* =  5, 11, 11 respectively); (B) ΔABR in 28 d treatment with SU-11248 (+/+, sham *Jbo/+*, drug *Jbo/+ n* = 10, 15, 15 respectively); (C) ΔABR in 21 d treatment with 50 mg/kg PTK787 (+/+, sham *Jbo/+*, drug *Jbo/+ n* = 9, 13, 15 respectively); (D) ΔABR in 28 d treatment with 75 mg/kg PTK787 (+/+, sham *Jbo/+*, drug *Jbo/+ n* = 40, 60, 40 respectively); (E) ΔABR in 28 d treatment with 17-DMAG (+/+, sham *Jbo/+*, drug *Jbo/+ n* = 9, 15, 15 respectively). In each experiment, the response to drug treatment was compared to the sham control. Histogram bars are mean ± SEM. Statistics were conducted using 1-tailed Mann Whitney U tests.

To investigate whether Vegf has a pro-inflammatory role in OM we employed a variety of small-molecule inhibitors of VEGFR and assessed their effects on OM when delivered systemically to the *Junbo* mouse mutant. The rationale for using the *Junbo* model and not *Jeff* was that the OM phenotype was more penetrant. The percentage of *Jbo/+* mice with bilateral OM was higher at 78% versus 46% in *Jf/+* (Figure S4) making auditory brainstem response (ABR) measurements more robust (see below). Moreover, hearing loss over the standard test period from day 28 to day 56 was greater in *Jbo/+* (averaging 7–14 dB in independent experiments) than in *Jf/+* (∼4 dB) (Figure 4 and Figure S5). When *Jbo/+* mice were treated with VEGFR signaling inhibitors BAY 43-9006 (30 mg/kg), SU-11248 (20 mg/kg) and PTK787/ZK 222584 (50 mg/kg or 75 mg/kg) (hereafter referred to as PTK787) there was a significant moderation of hearing loss (Figure 4). The trial with BAY 43-9006 was terminated after 2 wk when mice suddenly became piloerect. Although BAY 43-9006 was not as well tolerated as PTK787 and SU-11248, the positive therapeutic response to three separate VEGFR signaling inhibitors confirms our data, indicating that HIF mediated VEGF is a critical pathway in OM pathogenesis. We also proceeded to target HIF signaling directly using a HSP90 inhibitor, 17-DMAG. HSP90 is a chaperone of HIF-1α. We found that its use also moderated hearing loss (Figure 4).

**Figure 5:**
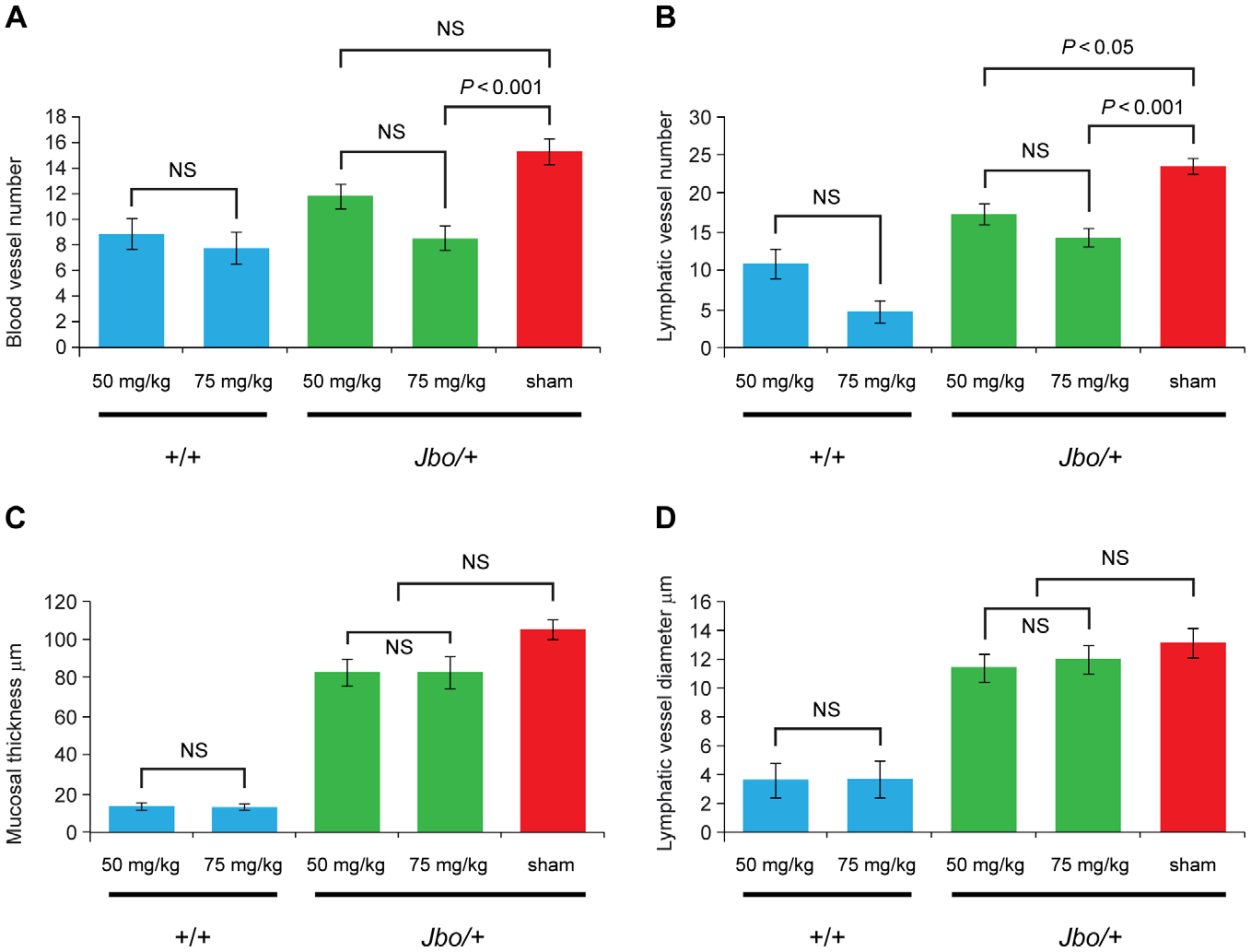
Treatment of *Jbo/+* mice with PTK787 reduces angiogenesis and lymphangiogenesis in the inflamed middle ear mucosa. *Junbo* (+/+ and *Jbo/+*) mice were treated with either 50 mg/kg or 75 mg/kg of PTK787 for 4 wk, the sham control *Jbo/+* groups received vehicle alone. The middle ear mucosa in treated *Jbo/+* mice had (A) fewer blood vessels in the 75 mg/kg treatment group and (B) fewer lymphatic vessels in the 50 mg/kg or 75 mg/kg treatment groups. (C) The mucosa thickness and (D) mucosal lymphatic vessel diameter did not differ significantly from sham treated controls. The +/+ group sizes were *n* = 8; *Jbo/+* drug treatment groups *n* = 15; *Jbo/+* sham group *n* = 28. Histogram bars represent mean ± SEM. Data in panels A, B and C were analyzed by one-way ANOVAs and Bonferroni's multiple comparison tests for post hoc testing. Lymphatic vessel number (panel B) was not normally distributed and a Kruskall-Wallis test was performed followed by Dunn's multiple comparison tests for post hoc testing.

We went on to examine the middle ear mucosal changes in mice treated with VEGFR inhibitors. Morphometric analysis of the mucosal histology was performed on 50 mg/kg and 75 mg/kg PTK787 treatment groups (Figure 5). ANOVA analyses revealed significant reductions in blood vessel number at the higher 75 mg/kg dose; lymphatic vessel number was reduced at both dosages; but neither the mucosal thickness nor lymphatic vessel diameter was reduced by PTK787 treatment (Figure 5). In the BAY 43-9006 trial, treated *Jbo/+* mice had reduced lymphatic vessel number (10.8±1.1 *n* = 11 mice versus 16.4±1.0 *n* = 11, *P* = 0.0012) and lymphatic vessel dilation (9.9±1.0 µm *n* = 11 versus 14.2±0.9 µm *n* = 11, *P* = 0.0072) compared with sham treated controls but mucosal thickness and blood vessel number were not altered.

To qualitatively assess the effect of drug treatment on bulla fluid accumulation, the middle ears were sampled in the 75 mg/kg PTK787, SU-11248 and 17-DMAG treated and sham treated *Jbo/+* mice. In each trial, a significantly lower (*P*<0.05) proportion of treated *Jbo/+* mice yielded bulla fluid samples than sham treated *Jbo/+* controls (Figure S6).

## Discussion

Single gene mutations in mouse *Eya4*, *Tlr4*, *p73*, *MyD88*, *Fas*, *E2f4*, *Plg*, *Fbxo11* and *Evi1* give rise to chronic spontaneous OM phenotypes, in several cases as part of a spectrum of pleiotropic effects, and are candidate susceptibility genes for human OM. In human populations there are significant associations between OM and polymorphisms in *FBXO11*, *TLR4* and *PAI1*. However, the mechanisms and pathways by which these mutations result in chronic middle ear inflammatory disease are poorly understood. It has been proposed that they may act by a variety of different mechanisms including altered Eustachian tube function and reduced clearance of middle ear pathogens, dysregulation of innate immunity via TLR4/MyD88 pathways and dysregulation of anti-inflammatory mechanisms via TGF-β pathways [27]. We have analyzed the *Junbo* and *Jeff* mutants using a number of approaches, including transcriptional profiling and, in *Junbo* mice, small-molecule inhibitors to dissect the genetic pathways and pathophysiological processes leading to chronic OM.

The characteristic lesion of OM is the accumulation of fluid and inflammatory cells in the bulla and mucosal inflammation. At other sites of inflammation, hypoxia is likely to occur as a result of the uptake of oxygen by inflammatory cells coupled with their physical separation from an underlying vascular bed [30]. Using PIMO labeling we have identified cellular hypoxia in inflammatory cells in the purulent *Jbo/+* and serous *Jf/+* fluids that accumulate within the 5–6 µl bulla [31]. However mucosal hypoxia was only detectable in *Jbo/+* mice. The driver of mucosal hypoxia may be the unmet oxygen demand of inflammatory cells in bulla fluids which in turn is presumably a function of their numbers and viability. The cellularity of *Jbo/+* bulla fluids is certainly >100-fold higher than in *Jf/+* mice but there are substantial apoptotic PMN cell populations (ranging from 20–40%) and a necrotic cell population (7–8%) which may affect overall oxygen requirements (Table 1). Apoptosis/cell death pathways and oxidative stress pathways would be expected to be upregulated as part of the inflammatory process. Human chronic OME effusions (with or without bacterial infection) range from purulent to serous and mucoid and contain viable and degenerative inflammatory cells [32], [33] and VEGF protein [21] which is a critical downstream mediator of hypoxia signaling. Our results provide direct evidence of cellular hypoxia in bulla fluid inflammatory cells whereas the data for pO_2_ in human OME bulla fluids is inconsistent [14]–[16]. Mucosal gas exchange is the main method of ventilation of the normal tubotympanum and the resting oxygen tension of the middle ear is comparable to that of venous blood [34]. Surgical ventilation causes relative hyperoxia of the middle ear [35] and a change in oxygen tension might also be an important mechanism in the down-regulation of HIF signaling. One therapeutic benefit may be reduced mucin secretion as conserved promoter regions of respiratory mucin genes expressed in human middle ear bind to HIF-1α [36], [37].

While the influx of inflammatory cells into the bulla lumen may be a key event in the development of hypoxia and activation of HIF signaling via stabilization of HIF-1α protein the activation of inflammatory cells and upregulation of Il-1β, Tnf-α and Nfκb in particular may further modulate HIF signaling [5], [6]. Transcriptional profiling showed upregulation of inflammatory gene networks in the bulla fluids of *Jbo/+* and *Jf/+* mice relative to blood WBC. Il-1β and Tnf-α serum titers are comparable in mutant and +/+ mice suggesting that OM is not a cause of systemic inflammation, nor is it part of an ongoing systemic inflammatory condition in *Junbo* and *Jeff* models. A number of inflammatory genes associated with OM have been published; for a review, see [38] and [39]–[63] and our array data adds another 20 genes to this list (Table S2). However, middle ear inflammation appeared less pronounced in *Jf/+* mice. In line with *Jf/+* serous bulla fluids containing fewer inflammatory cells, protein titers for the key cytokines Il-1β and Tnf-α were only elevated in a minority of mice. This degree of biological variation between individual *Jf/+* mice may explain the variability between pooled samples in which elevated expression levels for genes such as *Il-1β* and *Tnf-α* failed to achieve statistical significance. The relative contributions of Il-1β and Tnf-α to hypoxic modulation of Hif-1α and Vegf signaling in the middle ear may be greater in *Jbo/+* mice. Nevertheless in both mutants upregulation of HIF signaling was evident from the elevated expression of multiple Vegf signaling pathway genes (Figure S1 and Table S1) including Vegfa and in *Jbo/+* its principle receptor Kdr (Vegfr2). Elevated *Vegfa* gene expression was accompanied by elevated Vegfa protein in *Jbo/+* and *Jf/+* mice (Figure 2).

VEGF acts to induce angiogenesis, increases vascular permeability and recruitment of neutrophils and macrophages [64], [65] and may therefore contribute to OM by the accumulation of fluid and inflammatory cells within the bulla causing conductive hearing loss and secondary cochlear dysfunction via diffusion of cytokines through the round window [66], [67]. We tested the hypothesis that VEGF signaling contributes to OM pathogenesis by treating *Junbo* mice, which have highly penetrant OM, with the VEGFR signaling inhibitors PTK787, SU-11248, and BAY 43-9006 and the HSP90 inhibitor 17-DMAG. Their use reduced hearing loss (Figure 4). Histological analysis of the middle ear mucosa in PTK787 treated *Jbo/+* mice revealed reduced blood vessel formation (at the higher 75 mg/kg dose) and lymphatic vessel formation (at 50 mg/kg and 75 mg/kg dosages) consistent with the anti-angiogenic effects of VEGFR signaling inhibitors (Figure 5). Only lymphatic vessel number and diameter were significantly moderated by BAY 43-9006 but this may be a reflection of the initial acute inflammatory change taking place in the first 2 wk which was the end point of this trial. SU-11248 treated mice were not examined by histology.

Another effect of treatment with PTK787, SU-11248 and 17-DMAG was to reduce the proportion of *Jbo/+* mice that yielded bulla fluid samples (Figure S6). This may reflect moderation of VEGF induced vascular permeability in treated mice. The implication is that bulla fluids recoverable from treated *Jbo/+* mice come from those which are less responsive to treatment, and this would confound comparisons of inflammatory cell numbers and gene expression between treated and control mice.

The range of molecular targets for VEGFR and HSP90 inhibitors will require further clarification. VEGF receptors are members of the Receptor Tyrosine Kinase (RTK) superfamily and small-molecule VEGFR inhibitors have multi-kinase inhibitor profiles against different VEGF receptors as well as other RTK families. PTK787 is an inhibitor of VEGFR1, VEGFR2, VEGFR3, PDGFR-β and c-Kit; SU-11248 acts as a VEGFR2, PDGFR-β, FLT3 and c-Kit inhibitor; and BAY 43-9006 acts as a VEGFR2, FLT3, PDGFR-β, c-Kit and Raf1 inhibitor [68]. VEGFR inhibitors therefore have the potential to disrupt additional pathways [69] that might contribute to OM pathogenesis. We therefore also targeted HIF-VEGF signaling pathways using 17-DMAG treatment to inhibit HSP90. HSP90 chaperones a number of proteins involved in HIF-VEGF signaling including HIF-1α itself, the mitogenic signaling protein AKT, and RAF-1 in the RAS/RAF/MEK/ERK MAPK pathway [70]–[72]. In addition, phosphorylation of HSP90 by its client protein VEGFR2 is required for receptor signaling to endothelial NO synthase [73]. However, 17-DMAG can also attenuate inflammatory pathways [74] and may also contribute to the amelioration of OM observed in *Junbo* mice.

The expression of mutant *Evi1* and *Fbxo11* proteins in inflammatory cells in bulla fluids has the potential to perturb a variety of signaling pathways that may affect the response to hypoxia and contribute to OM pathogenesis. The *Fbxo11* gene is a member of the large F-box family which are specificity factors for the SCF E3 ubiquitin ligase complex, and in homozygote *Jeff* mutants there are developmental defects in palate, eyelid and lung airway as a result of perturbed Tgf-β signaling [75]. EVI1 is a co-transcriptional repressor of SMAD3 and the mutation in *Evi1* in *Junbo* mice may also exert effects via TGF-β signaling. EVI1 has two zinc-finger domains and a central transcription repression domain. Repressor activities via the proximal N-terminal zinc-finger domain include c-Jun N-terminal kinases (JNK) and TGF-β signaling via direct binding of SMAD3. SMAD3 activity is also reduced by recruitment of the co-repressor CtBP by the central repressor domain [76]. There is considerable cross-talk between TGF-β and HIF-1α pathways. For example, SMAD3 and HIF-1α are co-activators of VEGF expression [77], [78] and mutations affecting TGF-β signaling might be expected to perturb hypoxia responses.

The distal zinc-finger domain of EVI1 has three zinc-finger motifs [79] and the *Junbo* mutation is a non-conservative Asn763Ile change located within three amino acids of a contact residue in the second zinc-finger motif. Interactions with the distal zinc finger domain raise AP-1 activity by increased expression of *Jun* and *Fos* [80]. AP-1 and Jun also interact with HIF pathways [81], [82] and play a role in the pathogenesis of inflammatory bone and skin disease [83]. We found *Evi1*, and its target genes *Jun* and *Fos* were relatively upregulated in the bulla of both *Jbo/+* and *Jf/+* mice. However we cannot usefully speculate on the possibility of differential expression of *Jun* and *Fos* by mutant Evi1*^Jbo/+^* and wild type Evi1^+/+^ protein. Interpretation is problematic because bulla gene expression levels were normalized to their respective blood baselines, and we have no Evi1 protein data.

Our studies on the mutants *Junbo* and *Jeff*, highlights chronic inflammatory hypoxia as a key mechanism of OM pathogenesis and underlines the role of Hif-1α signaling in the underlying genetic and pathophysiological mechanisms that predispose to chronic OM. *Jeff* has a less pronounced inflammatory OM phenotype, nevertheless the underlying hypoxic signaling mechanism acting via VEGF appears similar to the *Junbo* model. As a consequence we have identified potential new therapeutic targets for OM. The practical clinical implications for using small-molecule VEGFR signaling inhibitors or other anti-VEGF agents and HSP90 inhibitors are limited in pediatric applications as they are used principally for the treatment of cancer [68], [69], [84]. Ototopical delivery appears to be the most likely way forwards to achieve therapeutic levels of small-molecule inhibitors in the bulla fluids whilst reducing any adverse effects caused by systemic administration. In summary, our findings on the genetic bases for OM in the *Junbo* and *Jeff* mutants have underlined the importance of hypoxia mechanisms in the development of chronic OM and as a consequence have revealed potential new therapeutic strategies that merit further exploration.

## Materials and Methods

### Ethics statement

The humane care and use of mice in this study was under the appropriate UK Home Office license.

### Mice

*Junbo* mice were congenic on a C3H/HeH background [29] and *Jeff* mice were on a mixed C3H/HeH and C57BL/6J genetic background [28]. The mice were specific pathogen free and had normal commensal nasopharyngeal flora [29].

### Blood and bulla fluid collection

Blood was collected from the retro-orbital sinus of mice under terminal anesthesia induced by an i.p. overdose of sodium pentobarbital. After removal of any adherent material on the external surface of the tympanic membrane, a hole in the membrane was made by removing the malleus with a clean pair of forceps and collecting the bulla fluid with a pipette. Bulla fluid volume was measured by collecting 0.5 µl aliquots and the total pooled samples from both ears generally ranged between 0.5–2.0 µl. Bulla fluid was collected into 100 µl aliquots of the appropriate buffer for each analysis (see below) or into 20 µl of RNase free water for RNA isolation. Whole blood for RNA isolation was collected in RNAlater (Qiagen).

### Hematology and bulla fluid analysis

Samples of bulla fluids from 8 wk old *Jbo/+* and *Jf/+* mice were analyzed for total WBC counts on an Advia 120 hematology analyzer (Bayer). Cytology preparations of bulla fluids were made on electrostatically charged slides (Superfrost Plus, Menzel Glaser), methanol fixed then stained with rat anti-mouse F4/80 Mab (MCA497) (AbD Serotech) and counterstained with haematoxylin.

### Pimonidazole labeling for hypoxia

*Jbo/+*, *Jf/+* and their respective wild type (+/+) controls were labeled 3 h *in vivo* by i.p. injection with 60 mg/kg pimonidazole (PIMO) (Hypoxyprobe, HPI Inc) dissolved in 100 µl of sterile PBS. For FACS, bulla fluid samples were collected into 100 µl aliquots of ice cold FACS buffer then stained with anti-PIMO FITC, anti-mouse Ly6G and Ly6C PerCP-Cy5.5 (BD Pharminogen) and anti-Annexin V Biotin (BD Pharminogen)/Streptavidin Pacific Blue (Invitrogen). Propidium iodide (BD Pharminogen) was used to assess necrotic cells. 50 µl EDTA blood samples were diluted in 100 µl FACS buffer then treated with RBC lysis buffer (BD Pharminogen). Unlabeled bulla fluid PMN and non-staining peripheral (normoxic) PMN from PIMO-labeled mice served as negative controls. For histology, the head with the tympanic membranes left intact was fixed for 48 h in 10% neutral buffered formalin then decalcified with Formical (Decal Corp) for 72 h. Wax embedded 3 µm dorsal plane sections of the middle ear were immunostained for PIMO or stained with haematoxylin and eosin.

### Real-time quantitative PCR (RT-qPCR) using Applied Biosystems' TaqMan assays

Total RNA from 4 independent pooled samples of *Jbo/+* and *Jf/+* bulla fluids was isolated using Nucleospin RNA/protein isolation kits (Macherey-Nagel). Individual blood samples from *Jbo/+* and *Jf/+* mice were extracted using Mouse RiboPure kits (Ambion) then the RNA was made into 3 separate sample pools. Each sample pool comprised 10–15 *Jbo/+* mice or 5–9 *Jf/+* mice. RNA quantity was measured on a Nanodrop 8000 (Thermo Fisher Scientific) and the integrity assessed by gel electrophoresis. 1 µg of RNA from each pool was used to synthesize double stranded cDNA with a High Capacity cDNA archive kit (AB).

RT-qPCR was performed using TaqMan gene expression assays using Fast Universal PCR Master Mix on a 7500 Fast Real-Time PCR System (AB). Three technical replicates were performed for each TaqMan assay. Data was normalized using Ppia as the endogenous control and fold changes of expression (ddCts) of bulla fluid WBC over blood WBC were calculated using AB 7500 software v2.0.1. This software allowed us to average the technical replicates for each pool and then average the biological replicates for the *n* = 4 bulla fluid sample pools and *n* = 3 blood sample pools. The fold change data is shown by mean relative quantification (RQ) ± min/max error bars representing 95% Confidence Limits (CL).

### Real-time quantitative PCR (RT-qPCR) using SABiosciences' RT^2^ Profiler PCR array system

Using the biological replicate pools of bulla fluids and bloods described above, Vegf signaling (PAMM-091c) and Inflammation Response and Autoimmunity (PAMM-077c) arrays (RT^2^-qPCT™, SA Biosciences) were performed. For each plate, 0.5 µg of RNA was converted to double stranded cDNA using the RT^2^ first strand synthesis kit. After mixing with the SABiosciences RT^2^ qPCR mastermix, the cDNA was pipetted into the 96 well profile plate and run on a 7500 Fast Real-Time PCR System (AB). Data was normalized using β-actin as an endogenous control and fold changes of expression of bulla fluid WBC over blood WBC were calculated using SA Biosciences online software (http://pcrdataanalysis.sabiosciences.com/pcr/arrayanalysis.php). The significance of the fold change is shown as a *P* value based on a Student's t-test of the replicate 2∧(−dCt) values for each gene in the *n* = 3 control blood and *n* = 4 bulla fluid sample pools.

### Vegfa, Il-1β, and Tnf-α protein assays

Blood was collected into serum-gel clotting activator tubes (Sarstedt). Measured volumes of bulla fluid were added to 100 µl of ice cold PBS, then vortexed and centrifuged at 500×g for 5 min at 4°C. Bulla fluid supernatants and serum samples were stored at −80°C until assay using Quantikine mouse Vegfa, Il-1β and Tnf-α ELISA kits (R&D Systems). Some serum and bulla fluid samples had cytokine titers beneath the lowest assay standard (23 pg/ml for Tnf-α and 12 pg/ml for Il-1β) and according to the manufacturer's instructions these results are not reportable.

### Drug treatment and auditory brain-stem response (ABR)

27–29 d old +/+ and *Jbo/+* mice were dosed by oral gavage once a day with 30 mg/kg BAY 43-9006, 20 mg/kg SU-11248, 50 or 75 mg/kg PTK787, or 10 mg/kg 17-dimethylaminoethylamino-17-demethoxy-geldanamycin (17-DMAG). +/+ mice were treated with drug as a control for unforeseen ototoxicity. DMSO stock solutions of BAY 43-9006 and SU-11248 (LC Laboratories) or aqueous solutions of PTK787 and 17-DMAG were frozen at −20°C then diluted 10-fold in 2% methyl cellulose for administration. Drug and sham *Jbo/+* groups were matched for age, gender and pre-trial ABR threshold (range 30–60 dB) and the sham group received vehicle alone. The anesthetized mouse was placed in right lateral recumbency with the speaker positioned 1.5 cm from the right ear, and a click-evoked ABR performed [85]. ABR measurements were made one day before the first treatment and one day after the last treatment. For ABR with recovery, anesthesia was induced by i.p. injection with a mixture of 10 mg/kg xylazine and 100 mg/kg ketamine and was reversed by 5 mg/kg atipamezole hydrochloride.

To assess whether treatment with SU-11248, 75 mg/kg PTK787, or 17-DMAG treatment altered bulla fluid accumulation a note was made whether fluids were recoverable.

### Histology

Middle ear histology was assessed in the BAY 43-9006, and the 50 or 75 mg/kg PTK787 trial mice. Morphometric evaluation was by blinded assessment of a standard 1000 µm length of middle ear mucosa (avoiding the cochlea and the region close to the Eustachian tube), the mucosal thickness was averaged from 5 measurements, and the numbers of capillaries and lymphatic vessels (and their diameter) were recorded.

### Statistics

D'Agostino & Pearson omnibus normality tests were performed on PTK787 histology data and Vegfa titer data. Blood vessel number, lymphatic vessel diameter and mucosal thickness were normally distributed and this data was subsequently analyzed using one-way ANOVAs and Bonferroni's multiple comparison tests for post hoc testing. Lymphatic vessel number and Vegfa titers were not normally distributed and a Kruskall-Wallis test was performed followed by Dunn's multiple comparison tests for post hoc testing. Arcsine transformed proportion data from FACS was analyzed using Student t-tests. Chi-squared tests were used to analyze tympanic membrane appearance (cloudy or clear) and the presence or absence of bulla fluids. All other data including ABR measurements (where interval data was in 5 dB increments) was analyzed using Mann Whitney U tests. In the drug trials, 1-tailed tests were used to test positive response to therapy, otherwise 2-tailed tests were used and values *P*<0.05 were considered significant. Data are presented as mean ± SEM (*n*) or in the case of Vegfa, Tnf-α and Il-1β protein titers with box and whisker plots.

## Supporting Information

Figure S1 Gene expression in 8 wk *Jbo/+* and *Jf/+* bulla fluid inflammatory cells compared with blood WBC. Relative Quantification (RQ) of gene expression using TaqMan RT-qPCR for (A) *Jbo/+* and (B) *Jf/+* mice. Data represents mean RQ ± min/max 95% CL, *n* = 3 blood and *n* = 4 bulla fluid sample pools.
(TIF)

Figure S2 Vegf pathway gene expression in 8 wk *Jbo/+* and *Jf/+* bulla fluid inflammatory cells compared with blood WBC. Gene expression: 1. 44% elevated in both *Jbo/+* and *Jf/+* (>2 fold, *P*<0.05). 2. 12% elevated in both but only *Jf/+ P*<0.05. 3. 6% elevated in both but only *Jbo/+ P*<0.05. 4. 4% elevated in both *Jbo/+* and *Jf/+* but neither achieve statistical significance. 5. 8% elevated in *Jbo/+* but undetected in *Jf/+*. 6. 11% elevated in one mutant but unaltered in the other. 7. 14% unaltered in both *Jbo/+* and *Jf/+*. 8. 1% lower in *Jbo/+* and *Jf/+*.
(TIF)

Figure S3 Inflammation pathway gene expression in 8 wk *Jbo/+* and *Jf/+* bulla fluid WBC compared with blood WBC. Gene expression: 1. 35% elevated in both *Jbo/+* and *Jf/+* (>2 fold, *P*<0.05). 2. 12% elevated in both but only *Jf/+ P*<0.05. 3. 19% elevated in both but only *Jbo/+ P*<0.05. 4. 11% elevated in both *Jbo/+* and *Jf/+* but neither achieve statistical significance. 5. 6% elevated in *Jbo/+* but undetected in *Jf/+*. 6. 10% elevated in one mutant but unaltered in the other. 7. 5% unaltered in both *Jbo/+* and *Jf/+*. 8. 1% elevated in *Jf/+* and lower in *Jbo/+*. 9. 2% unaltered in *Jbo/+* and lower in *Jf/+*.
(TIF)

Figure S4 The gross OM phenotype is more penetrant in *Jbo/+* than in *Jf/+* mice. (A) The cloudy appearance ear drum is a semi-quantitative measure of bulla fluid accumulation. Wild type (+/+) mice have clear eardrums and the malleus is easily recognizable, while affected *Jbo/+* and *Jf/+* mice have cloudy ear drums. (B) The proportion of *Jbo/+* mice with bilateral and unilateral eardrum cloudiness is greater than in *Jf/+* mice. *Jbo/+ n* = 54, *Jf/+ n* = 50. 2×3 contingency table Chi-square = 9.99, 2 df, 2-tailed *P* = 0.007. Nevertheless, the majority of *Jbo/+* and *Jf/+* mice without grossly evident fluid have some degree of microscopic OM.
(TIF)

Figure S5 The increase in ABR (dB) thresholds between day 28 and day 56 is greater in *Jbo/+* than *Jf/+* mice. Wild type (+/+) *Junbo* and +/+ *Jeff* mice have ABR thresholds of 20–30 dB range and thresholds do not rise significantly in the day 28 to day 56 interval. In both *Jbo/+* and *Jf/+* mice, the ABR thresholds at day 28 are elevated, but the rise is greater in *Jbo/+* than *Jf/+* mice. Because of the higher incidence of unilateral OM, ABRs were recorded from both ears in *Jeff* mice. Mean ± SEM, *n* = number of mice. Paired Mann Whitney 2-tailed tests.
(TIF)

Figure S6 The occurrence of fluid in bulla fluid is reduced in *Jbo/+* mice treated with VEGF receptor inhibitors and the HSP90 inhibitor 17-DMAG. 75 mg/kg PTK787 treated *n* = 40, sham *n* = 30. 2×2 contingency table Chi-square = 4.92, 2 df, 2-tailed *P* = 0.0265. 20 mg/kg SU-11248 treated *n* = 30, sham *n* = 30. Chi-square = 4.51, 2 df, 2-tailed *P* = 0.0338. 10 mg/kg 17-DMAG treated *n* = 30, sham *n* = 30. Chi-square = 4.31, 2 df, 2-tailed *P* = 0.0379.
(TIF)

Table S1 Vegf pathway gene expression in 8 wk *Jbo/+* and *Jf/+* bulla fluid inflammatory cells compared with blood WBC. Gene expression was determined using RT^2^-qPCR arrays (SA Biosciences). Fold-change is the normalized gene expression in the bulla fluid sample divided by the normalized gene expression in the control blood sample. Data represents mean fold-change with values >2 indicated in red and those <−2 indicated in blue. *P* values are based on a Student's t-test of the replicate 2∧(− Delta Ct) values for *n* = 3 blood and *n* = 4 bulla fluid sample pools.A: This gene's average threshold cycle is relatively high (>30) in either the control or the test sample, and is reasonably low in the other sample (<30). B: This gene's average threshold cycle is relatively high (>30), meaning that its relative expression level is low, in both control and test samples. C: This gene's average threshold cycle is either not determined or greater than the defined cut-off value (default 35), in both samples meaning that its expression was undetected, making this fold-change result erroneous and un-interpretable.
(XLS)

Table S2 Inflammatory gene expression in 8 wk *Jbo/+* and *Jf/+* bulla fluid inflammatory cells compared with blood WBC. References are given to previously published genes that are modulated in otitis media. *Genes that are upregulated in both mutants that have not been previously associated in the literature with OM.
(XLS)
