# Supplementary figures and images for: HIF–VEGF Pathways Are Critical for Chronic Otitis Media in Junbo and Jeff Mouse Mutants
Source: PLoS Genet. 2011 Oct 20;7(10):e1002336. doi: 10.1371/journal.pgen.1002336 (PMC3197687; doi:10.1371/journal.pgen.1002336)

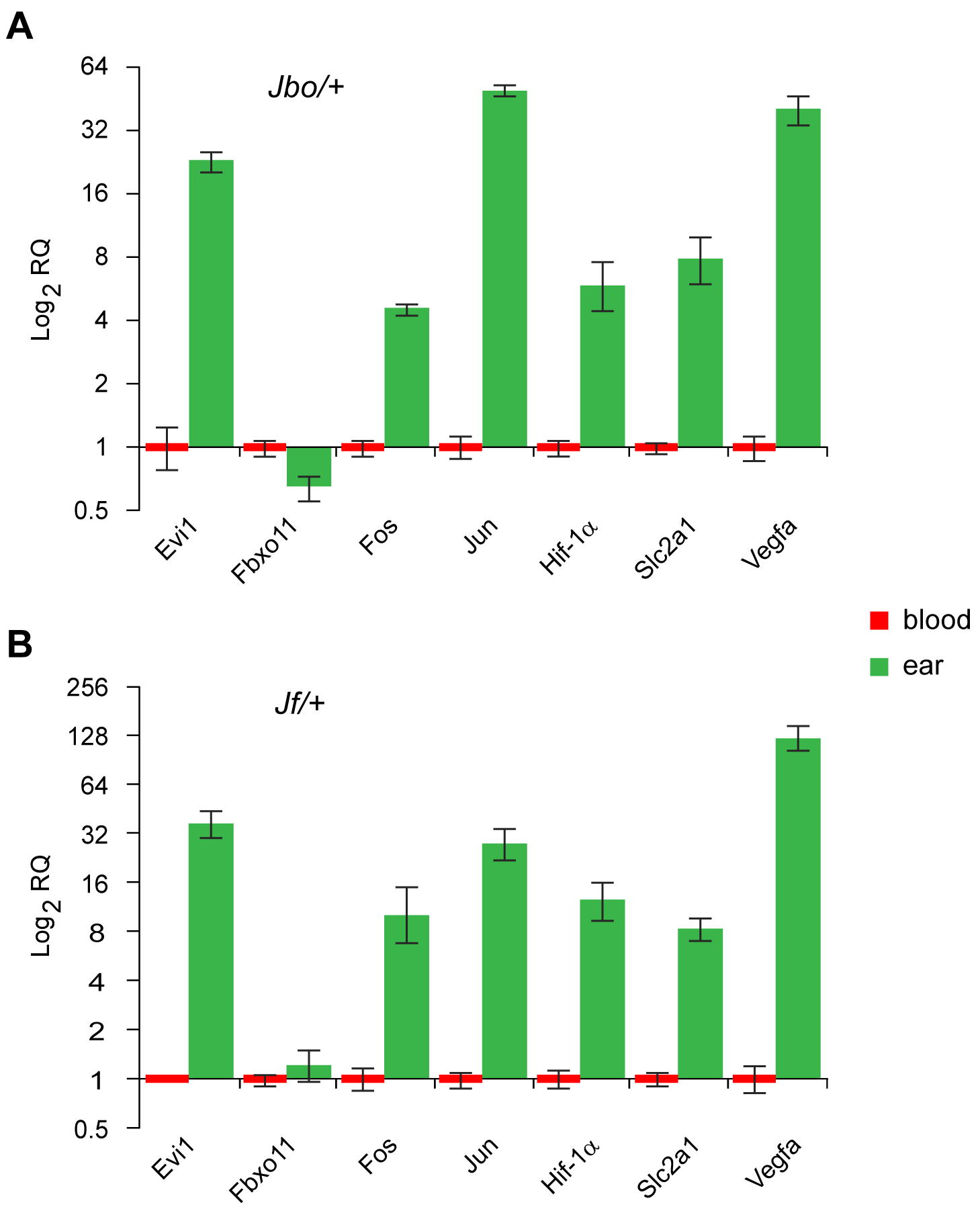

Supplement: Figure S1 — Gene expression in 8 wk Jbo/+ and Jf/+ bulla fluid inflammatory cells compared with blood WBC. Relative Quantification (RQ) of gene expression using TaqMan RT-qPCR for (A) Jbo/+ and (B) Jf/+ mice. Data represents mean RQ ± min/max 95% CL, n = 3 blood and n = 4 bulla fluid sample pools. (TIF) [file pgen.1002336.s001.tif]

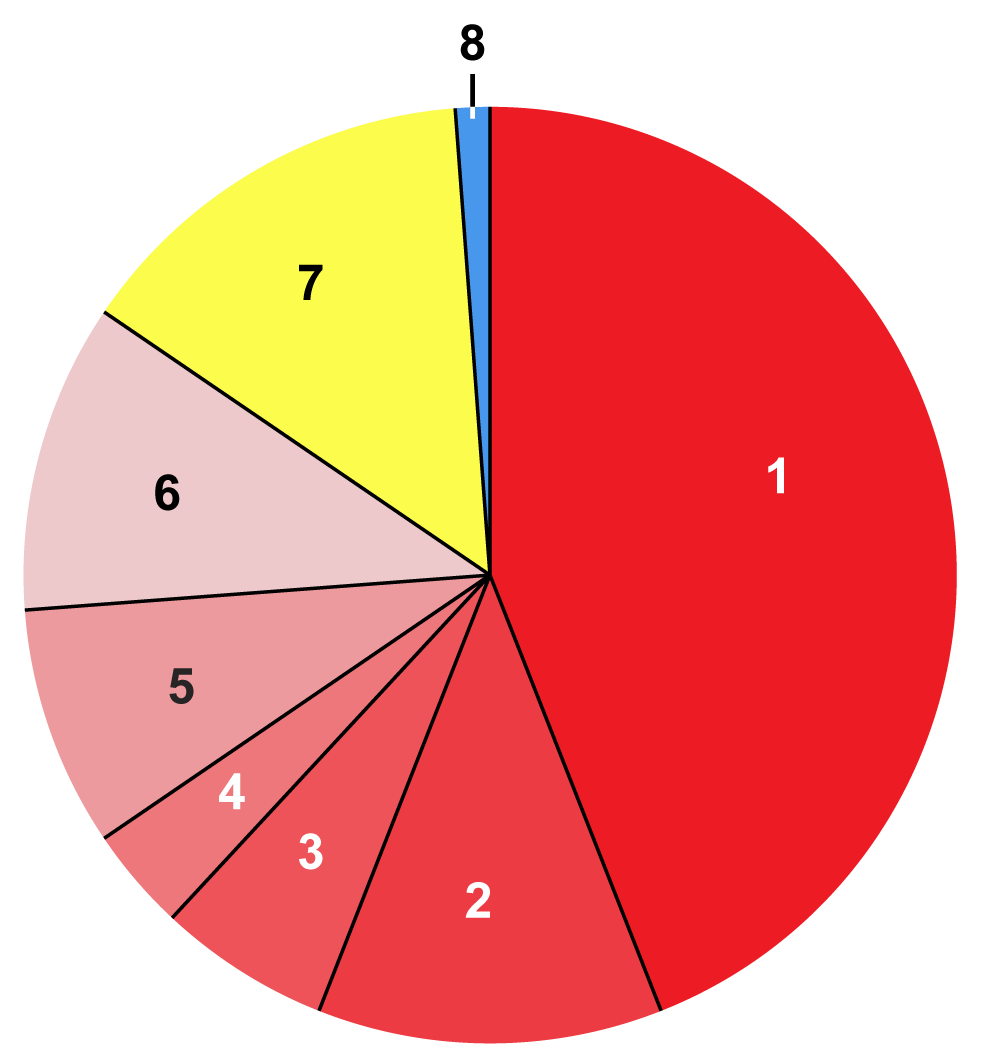

Supplement: Figure S2 — Vegf pathway gene expression in 8 wk Jbo/+ and Jf/+ bulla fluid inflammatory cells compared with blood WBC. Gene expression: 1. 44% elevated in both Jbo/+ and Jf/+ (>2 fold, P<0.05). 2. 12% elevated in both but only Jf/+ P<0.05. 3. 6% elevated in both but only Jbo/+ P<0.05. 4. 4% elevated in both Jbo/+ and Jf/+ but neither achieve statistical significance. 5. 8% elevated in Jbo/+ but undetected in Jf/+. 6. 11% elevated in one mutant but unaltered in the other. 7. 14% unaltered in both Jbo/+ and Jf/+. 8. 1% lower in Jbo/+ and Jf/+. (TIF) [file pgen.1002336.s002.tif]

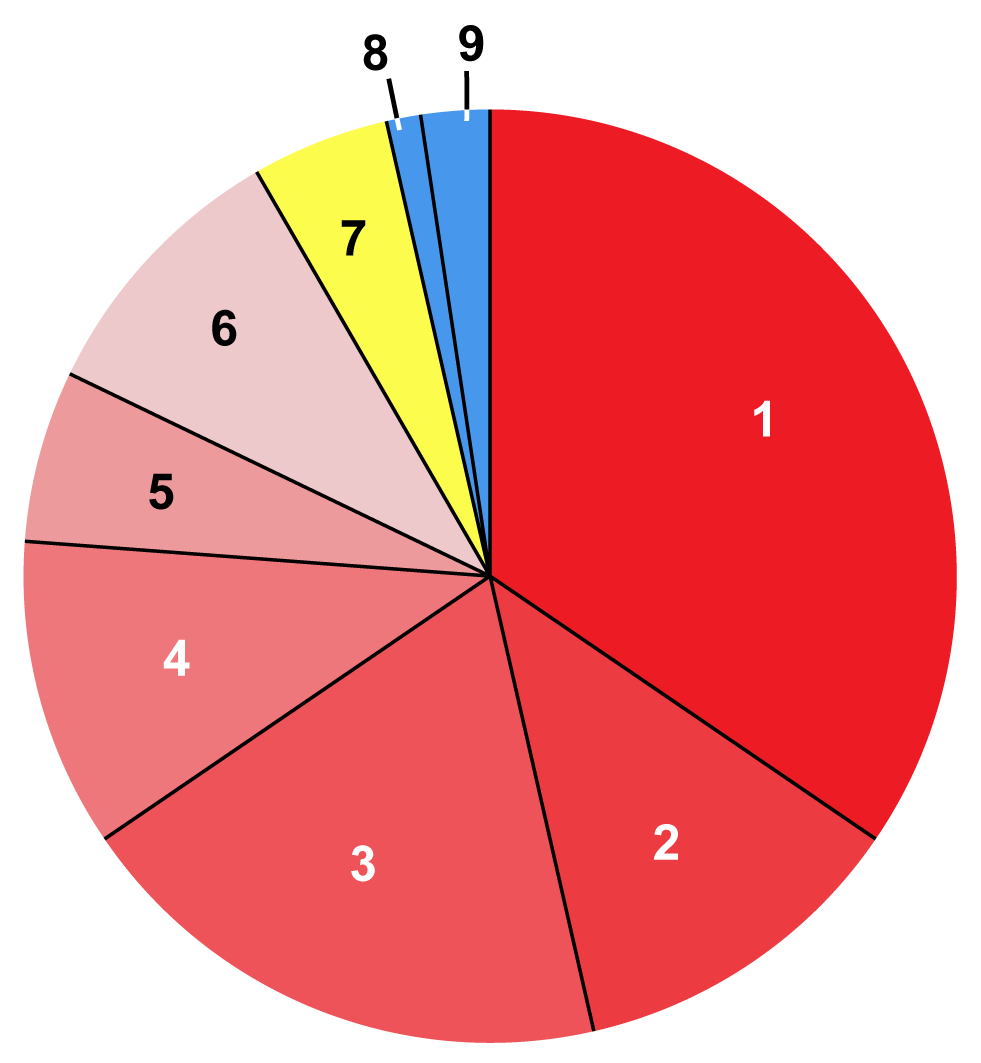

Supplement: Figure S3 — Inflammation pathway gene expression in 8 wk Jbo/+ and Jf/+ bulla fluid WBC compared with blood WBC. Gene expression: 1. 35% elevated in both Jbo/+ and Jf/+ (>2 fold, P<0.05). 2. 12% elevated in both but only Jf/+ P<0.05. 3. 19% elevated in both but only Jbo/+ P<0.05. 4. 11% elevated in both Jbo/+ and Jf/+ but neither achieve statistical significance. 5. 6% elevated in Jbo/+ but undetected in Jf/+. 6. 10% elevated in one mutant but unaltered in the other. 7. 5% unaltered in both Jbo/+ and Jf/+. 8. 1% elevated in Jf/+ and lower in Jbo/+. 9. 2% unaltered in Jbo/+ and lower in Jf/+. (TIF) [file pgen.1002336.s003.tif]

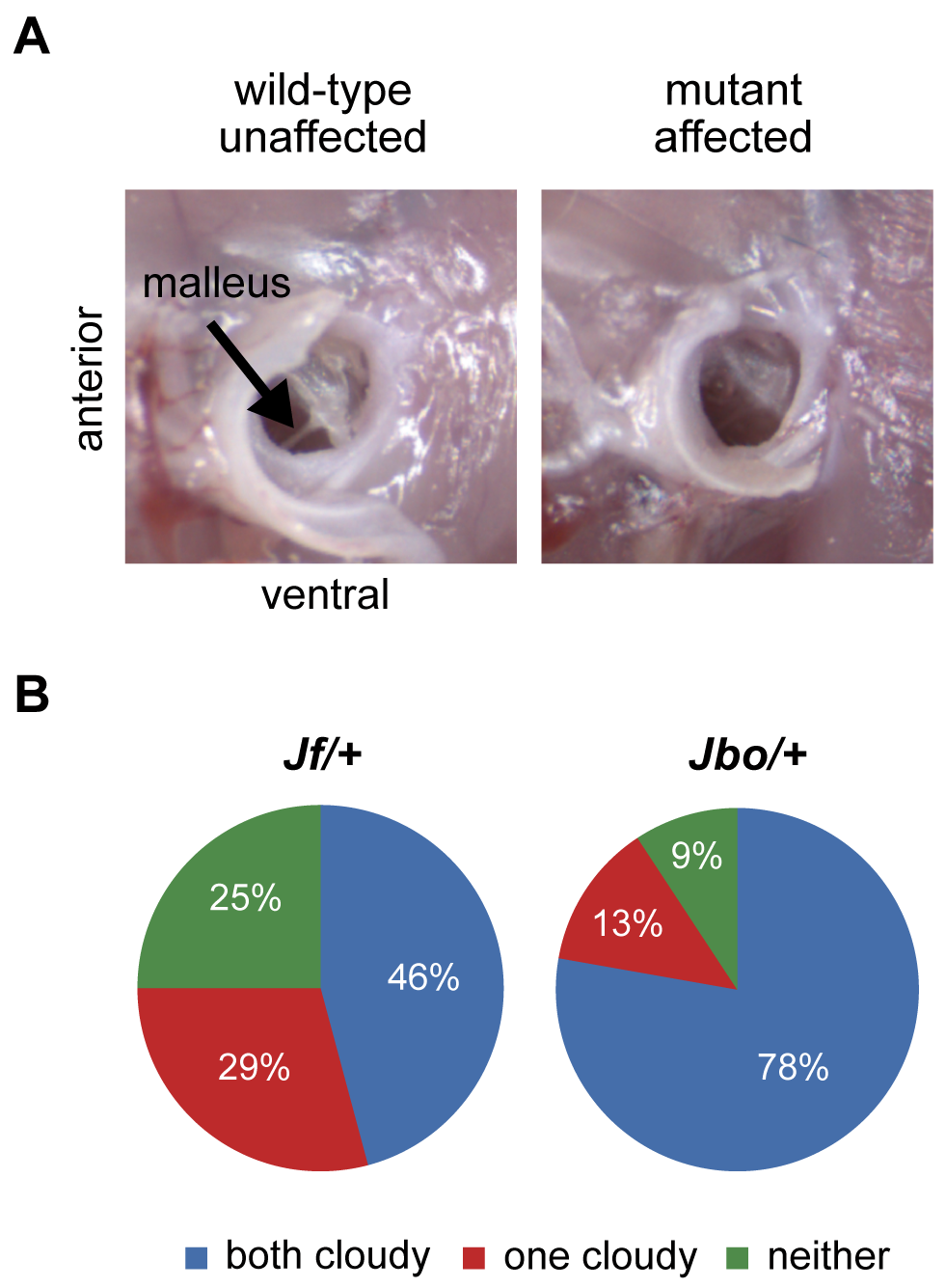

Supplement: Figure S4 — The gross OM phenotype is more penetrant in Jbo/+ than in Jf/+ mice. (A) The cloudy appearance ear drum is a semi-quantitative measure of bulla fluid accumulation. Wild type (+/+) mice have clear eardrums and the malleus is easily recognizable, while affected Jbo/+ and Jf/+ mice have cloudy ear drums. (B) The proportion of Jbo/+ mice with bilateral and unilateral eardrum cloudiness is greater than in Jf/+ mice. Jbo/+ n = 54, Jf/+ n = 50. 2×3 contingency table Chi-square = 9.99, 2 df, 2-tailed P = 0.007. Nevertheless, the majority of Jbo/+ and Jf/+ mice without grossly evident fluid have some degree of microscopic OM. (TIF) [file pgen.1002336.s004.tif]

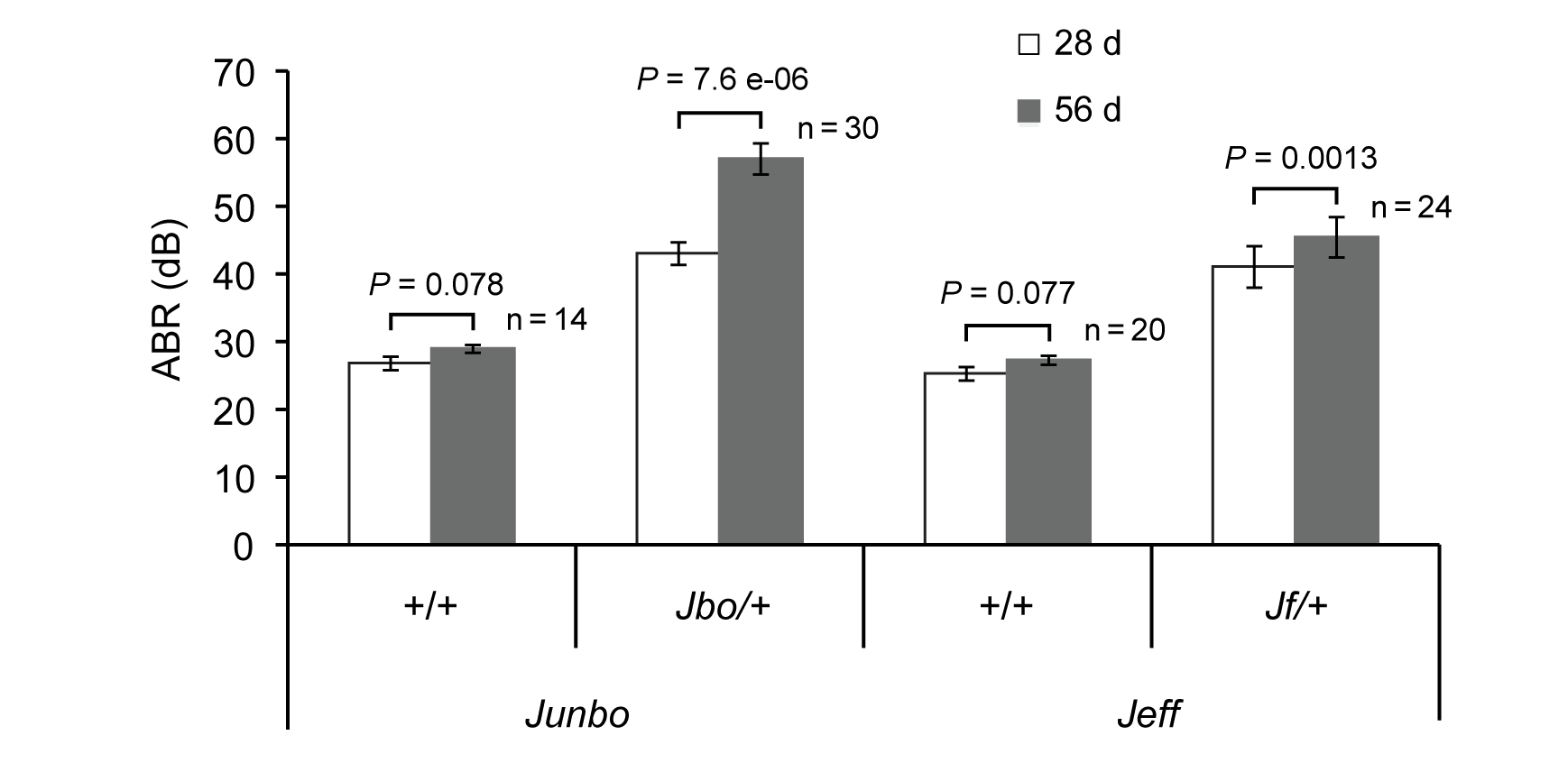

Supplement: Figure S5 — The increase in ABR (dB) thresholds between day 28 and day 56 is greater in Jbo/+ than Jf/+ mice. Wild type (+/+) Junbo and +/+ Jeff mice have ABR thresholds of 20–30 dB range and thresholds do not rise significantly in the day 28 to day 56 interval. In both Jbo/+ and Jf/+ mice, the ABR thresholds at day 28 are elevated, but the rise is greater in Jbo/+ than Jf/+ mice. Because of the higher incidence of unilateral OM, ABRs were recorded from both ears in Jeff mice. Mean ± SEM, n = number of mice. Paired Mann Whitney 2-tailed tests. (TIF) [file pgen.1002336.s005.tif]

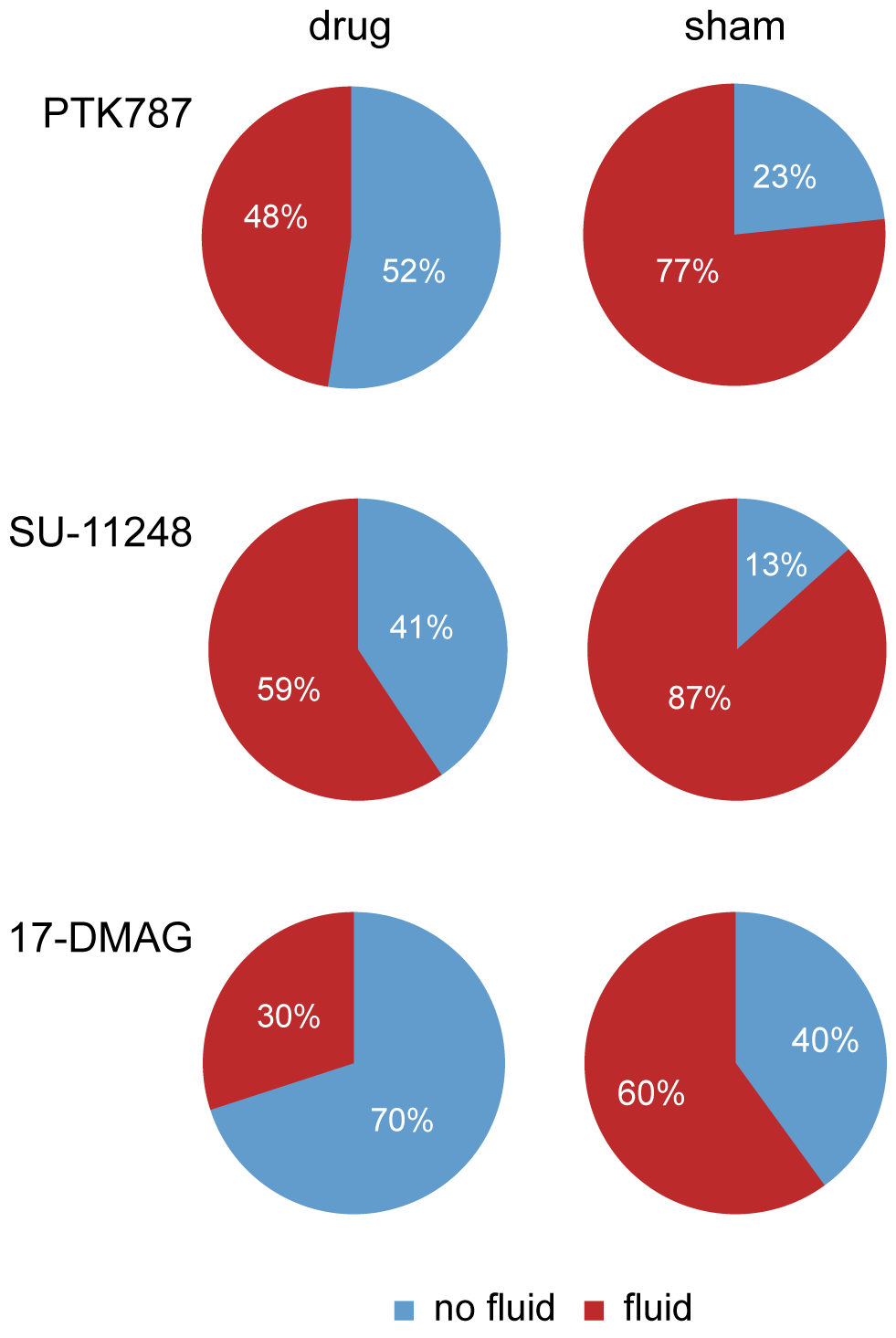

Supplement: Figure S6 — The occurrence of fluid in bulla fluid is reduced in Jbo/+ mice treated with VEGF receptor inhibitors and the HSP90 inhibitor 17-DMAG. 75 mg/kg PTK787 treated n = 40, sham n = 30. 2×2 contingency table Chi-square = 4.92, 2 df, 2-tailed P = 0.0265. 20 mg/kg SU-11248 treated n = 30, sham n = 30. Chi-square = 4.51, 2 df, 2-tailed P = 0.0338. 10 mg/kg 17-DMAG treated n = 30, sham n = 30. Chi-square = 4.31, 2 df, 2-tailed P = 0.0379. (TIF) [file pgen.1002336.s006.tif]
